# MicroRNA-31 Emerges as a Predictive Biomarker of Pathological Response and Outcome in Locally Advanced Rectal Cancer

**DOI:** 10.3390/ijms17060878

**Published:** 2016-06-03

**Authors:** Cristina Caramés, Ion Cristobal, Víctor Moreno, Juan P. Marín, Paula González-Alonso, Blanca Torrejón, Pablo Minguez, Ana Leon, José I. Martín, Roberto Hernández, Manuel Pedregal, María J. Martín, Delia Cortés, Damian García-Olmo, María J. Fernández, Federico Rojo, Jesús García-Foncillas

**Affiliations:** 1 Medical Oncology Department, Oncohealth Institute, Health Research Institute FJD-UAM, University Hospital Fundación Jiménez Díaz, Avda. Reyes Católicos-2, 28040 Madrid, Spain; ccarames@fjd.es (C.C.); victor.moreno@fjd.es (V.M.); Aleon@fjd.es (A.L.); JIMartin@fjd.es (J.I.M.); rhernandezl@quironsalud.es (R.H.); manuel.pedregal@quironsalud.es (M.P.); 2 Traslational Oncology Division, Oncohealth Institute, Health Research Institute FJD-UAM, University Hospital Fundación Jiménez Díaz, Avda. Reyes Católicos-2, 28040 Madrid, Spain; blanca.torrejon@quironsalud.es; 3 Radiation Oncology Department, University Hospital Fundación Jiménez Díaz, Avda. Reyes Católicos-2, 28040 Madrid, Spain; marin5si@yahoo.com; 4 Pathology Department, Health Research Institute FJD-UAM, University Hospital Fundación Jiménez Díaz, Avda. Reyes Católicos-2, 28040 Madrid, Spain; paula.galonso@fjd.es (P.G.-A.); mgg10167@gmail.com (M.J.F.); frojo@fjd.es (F.R.); 5 Department of Genetics and Genomics, Health Research Institute FJD-UAM, University Hospital Fundación Jiménez Díaz, Avda. Reyes Católicos-2, 28040 Madrid, Spain; pablo.minguez@quironsalud.es; 6 Gastroenterology Department, University Hospital Fundación Jiménez Díaz, Avda. Reyes Católicos-2, 28040 Madrid, Spain; MJMartin@fjd.es; 7 Surgery Department, Oncohealth Institute, Health Research Institute FJD-UAM, University Hospital Fundación Jiménez Díaz, Avda. Reyes Católicos-2, 28040 Madrid, Spain; DCORTES@fjd.es (D.C.); Damian.garcia@uam.es (D.G.-O.)

**Keywords:** miR-31, neoadjuvant chemoradiotherapy, rectal cancer, prognosis

## Abstract

Neoadjuvant chemoradiotherapy (CRT) followed by total mesorectal excision has emerged as the standard treatment for locally advanced rectal cancer (LARC) patients. However, many cases do not respond to neoadjuvant CRT, suffering unnecessary toxicities and surgery delays. Thus, identification of predictive biomarkers for neoadjuvant CRT is a current clinical need. In the present study, microRNA-31 expression was measured in formalin-fixed paraffin-embedded (FFPE) biopsies from 78 patients diagnosed with LARC who were treated with neoadjuvant CRT. Then, the obtained results were correlated with clinical and pathological characteristics and outcome. High microRNA-31 (miR-31) levels were found overexpressed in 34.2% of cases. Its overexpression significantly predicted poor pathological response (*p* = 0.018) and worse overall survival (OS) (*p* = 0.008). The odds ratio for no pathological response among patients with miR-31 overexpression was 0.18 (Confidence Interval = 0.06 to 0.57; *p* = 0.003). Multivariate analysis corroborated the clinical impact of miR-31 in determining pathological response to neoadjuvant CRT as well as OS. Altogether, miR-31 quantification emerges as a novel valuable clinical tool to predict both pathological response and outcome in LARC patients.

## 1. Introduction

Colorectal carcinoma (CRC) incidence, morbidity, and mortality rates vary markedly around the world, and rectal carcinoma represents approximately 28% of all CRC [1]. The standard of care for locally advanced rectal cancer (LARC) involves a multidisciplinary approach consisting of neoadjuvant chemoradiotherapy (CRT) followed by total mesorectal excision (TME) surgery. This fact was established after several studies showing that CRT before total mesorectal excision (neoadjuvant chemoradiotherapy) was associated with a lower percentage of patients with local recurrence than either with total mesorectal excision surgery followed by adjuvant CRT or total mesorectal excision surgery alone [2,3]. After the initiation of neoadjuvant CRT, a relevant observation is the range of pathological tumor response, from cases achieving a pathological complete response (pCR; ypT0N0) to others getting an absence of tumor regression at all or even tumor progression during therapy [4,5]. Of importance, pCR detected by pathological examination of the resected specimen is associated with significantly better prognosis as compared with patients with residual tumor, particularly residual nodal disease. Similarly, minimal residual disease is associated with better prognosis than gross residual disease [6,7]. Currently, the neoadjuvant CRT regimen most widely used is based on fluoropyrimidines concurrent with conventional fractionation radiotherapy [2]. Nevertheless, more than one-third of cases develop distant metastasis within 10 years from diagnosis and complete pathological response only occurs in 8% to 14% of patients. Thus, those patients who do not respond suffer undesired toxicities and delays in the resection of the primary tumor [8,9]. 

There are currently no effective methods to predict which patients will respond to neoadjuvant CRT. Despite some prognostic factors of local recurrence, distant recurrence and outcome have been proposed after tumor mesorectal excision surgery [10,11,12]; neoadjuvant CRT predictive biomarkers with an impact in recurrence and outcome are not still established in clinical practice [12,13,14]. The identification of patients who have a higher possibility of responding to preoperative CRT could be important in improving survival and decreasing treatment morbidity and local control in LARC. Moreover, patients who are potential non-responders could be moved to alternative therapeutic strategies. Therefore, the identification of novel alterations with predictive value of response to neoadjuvant CRT would be of high relevance for an optimal multidisciplinary treatment approach in this LARC patient subgroup.

MicroRNAs (miRNAs) are short (18–27 nucleotides) non-coding single-stranded RNA molecules which negatively regulate the expression of specific target genes at the post-transcriptional level. Depending on their target genes, microRNA deregulation has been related to CRC development, progression, or therapy response. Moreover, they can be easily detected in rectal cancer tissue and blood, so they have been proposed as promising biomarkers for diagnosis, prognosis, and monitoring therapies in this disease [15,16,17]. MicroRNA-31 (miR-31) maps on 9p21.3 and is one of the most significantly deregulated miRNAs in rectal cancer. It has been reported to be overexpressed in CRC cell lines as well as in rectal tumor tissue compared with the normal paired rectal mucosa [18]. In functional studies, the inhibition of miR-31 is able to impair CRC cell proliferation, invasion, and promote apoptosis—which suggests its oncogenic role in this disease [19]. Furthermore, high miR-31 levels have been described to correlate with tumor stage [18,20], poor prognosis [21], and shorter progression-free survival in CRC patients treated with anti-epithelial growth factor receptor (anti-EGFR) therapies [21]. Of importance, miR-31 suppression increases sensitivity to 5-fluorouracil (5-FU) and affects cell migration and invasion in the CRC cell line HCT-116 [22]. However, miR-31 ability to predict neoadjuvant CRT pathological response and outcome in LARC is a relevant question that remains to be explored. Here, we report that miR-31 deregulation is a common event in LARC that determines poor outcome. Moreover, this alteration is of high therapeutic relevance because it defines a subgroup of LARC patients who will not respond to neoadjuvant CRT.

## 2. Results

### 2.1. Prevalence of miR-31 Deregulation in Locally Advanced Rectal Cancer (LARC) and Its Relation with Pathological and Clinical Characteristics

**Figure 1:**
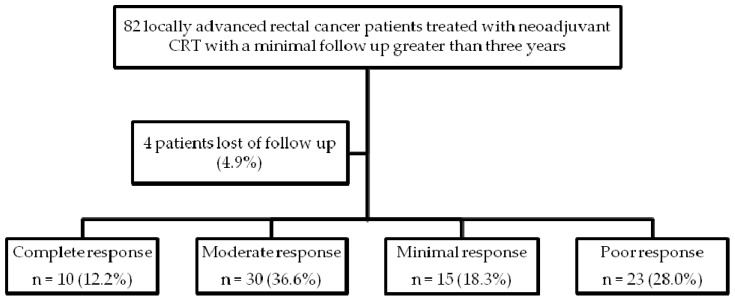
Patients flowchart through the study. CRT: chemoradiotherapy.

Eighty-two patients with LARC treated with neoadjuvant CRT were selected. All of them had a minimal follow-up greater than three years. The cohort flowchart diagram is shown in Figure 1. Owing to the loss of clinical follow-up data, four patients were excluded. Complete response, moderate response, minimal response, and a poor response was observed in 12.3%, 37%, 18.3%, and 28.4% of patients, respectively.

High miR-31 levels were observed in 34.2% of cases (27 out of 78). To explore the potential value of miR-31 predicting response to neoadjuvant CRT, patients with different grades of pathological response were compared. Interestingly, those patients who developed complete response tended to have low microRNA-31 levels, whereas the subgroup of patients with a poor pathological response showed high levels of miR-31. (*p* = 0.018). No statistical significance was observed in age, sex, Eastern Cooperative Oncology Group (ECOG) scale of performance status, histological grade, clinical stage, tumor size, and lymph node stage between patients with high and low miR-31 expression levels (Table 1). Similarly, no differences were found for pathological stage and downstaging.

### 2.2. miR-31 Deregulation Predicts Pathological Response to Neoadjuvant Chemoradiotherapy (CRT) in LARC Patients

**Figure 2:**
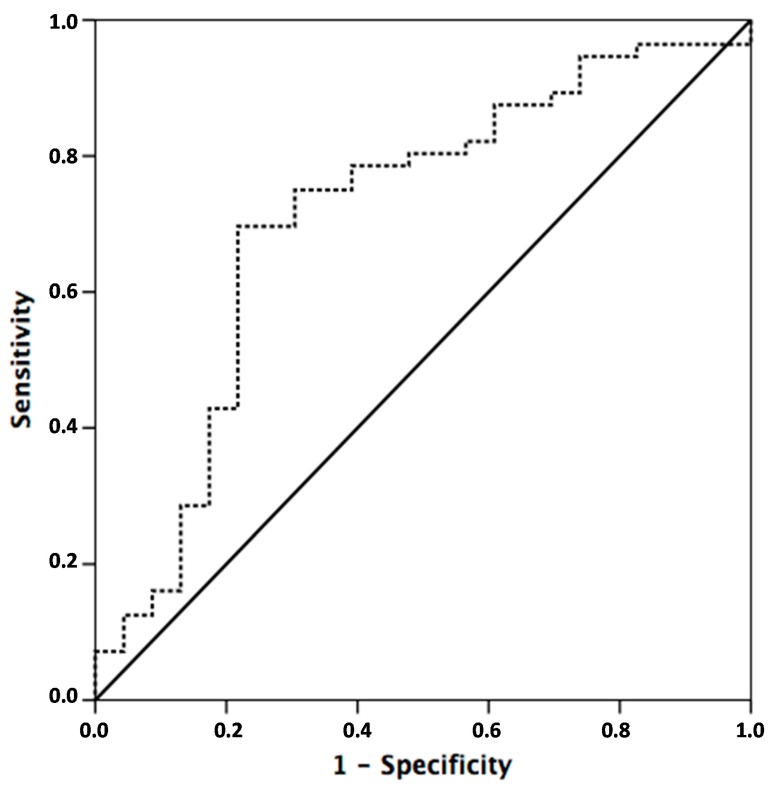
Receiver operating characteristic (ROC) Curve. Based on this ROC curve, the cutoff point that provides the best sensitivity and specificity to separate rectal cancer from any response to absence of response (Ryan 3) was selected. According with this criteria, miR-31 high (or overexpressed) was defined as Δ*C*_T_ miR-31 < 0.34 and miR-31 low was defined as Δ*C*_T_ miR-31 > 0.34. The dash line represents the coordinated points of the ROC curve. The solid line represents the ROC curve diagonal reference line.

Receiver operating characteristic (ROC) curves were generated in order to investigate the potential utility of miR-31 as a predictive biomarker of response to neoadjuvant CRT. miR-31 expression levels provide an area under the curve (AUC) value of 0.71 (95% confidence interval (CI) = 0.57 to 0.84; *p* = 0.001) with 60.8% sensitivity and 76.3% specificity in distinguishing rectal cancer from poor responders to minimal, moderate, and complete responders. Negative predictive value (NPV) was 82.3% and positive predictive value (PPV) was 51.8% (Figure 2 and Table 2).

Moreover, multivariable logistic regression analyses revealed that miR-31 expression levels measured before the beginning of neoadjuvant CRT is a predictive pathological response marker. The odds ratio for non-responders was 0.18 (95% CI = 0.06 to 0.57; *p* = 0.003) (Table 3).

### 2.3. High miR-31 Levels Determines Poor Outcome in LARC Patients Treated with Neoadjuvant CRT

**Figure 3:**
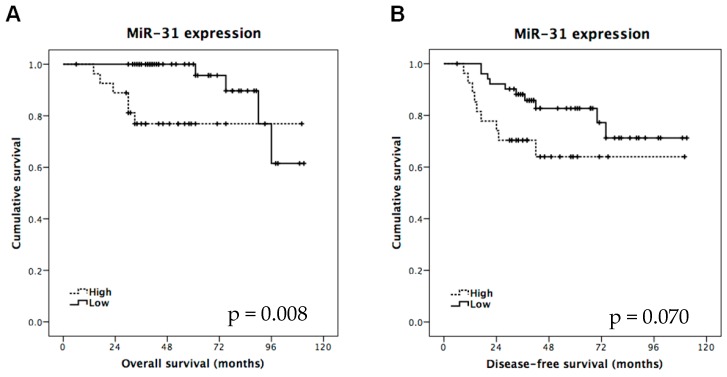
Kaplan-Meier analysis of (**A**) overall survival (OS) and (**B**) disease-free survival (DFS) in LARC patients based upon miR-31 expression in primary tumors measured prior to the neoadjuvant treatment.

To further evaluate the clinical significance of miR-31 in LARC, we next investigated whether miR-31 levels could serve as a predictor of patient outcome. Interestingly, patients with higher miR-31 levels had statistically significantly worse overall survival (*p* = 0.008, Figure 3A). Despite high miR-31 expression also associated with shorter disease-free survival, significance was not achieved (*p* = 0.070)—probably due to the small number of cases included in this cohort (Figure 3B).

Additionally, Cox proportional hazard regression analyses showed that in the univariate analysis, poor outcome in LARC patients was related to high levels of miR-31 (Hazard Ratio (HR) = 5.077; 95% CI = 1.366 to 18.863; *p* = 0.015) and pathological stage (HR = 1.890; 95% CI = 1.027 to 3.478; *p* = 0.043). In the same way, multivariable analysis proved that high levels of miR-31 can be used as an independent prognostic biomarker for determining outcome in LARC patients treated with neoadjuvant CRT (HR = 0.206; 95% CI = 0.051 to 0.840; *p* = 0.028) (Table 4).

## 3. Discussion

Personalized treatment selection for patients with LARC relies on clinical variables measured prior to neoadjuvant CRT. Taken into account that, depending on the series, between 45% and 20% of the patients do not respond to neoadjuvant CRT therapy, and the lack of any clinical and pathological factors to guide the initial treatment, novel predictive biomarkers are needed to avoid toxicity and surgery delays. In this context, prior studies have reported the potential usefulness of some gene signatures and microRNA expression profiles to predict neoadjuvant CRT response [14,17]. However, those biomarkers are not well validated for use in daily clinical practice. Here, we show that miR-31 expression levels measured before starting any treatment have the potential ability to predict pathological response, overall survival, and progression-free survival in patients with LARC treated with 5-FU based neoadjuvant CRT.

We decided to evaluate the potential clinical value of miR-31 in predicting CRT response due to the previously-reported oncogenic role of this microRNA in CRC [23]. We found a similar prevalence (around 34%) of miR-31 overexpression to what has been previously reported in the literature (between 10% and 38% depending on the cohort and the stage of the disease) [23,24]. In concordance with our findings, it has been reported that miR-31 suppression increments sensitivity to 5-FU at the initial stage, and also affects invasion and cell migration in HCT-116 cells [22]. Moreover, several studies have reported the potential value of microRNAs to predict neoadjuvant CRT response; however, these works did not evaluate miR-31 expression levels [25,26].

In our study, high miR-31 levels were significantly associated with the lack of pathological response and patients with low miR-31 expression tended to have better response. As indicated above, the pathological response can be predicted with a specificity of 76.3%. Prior works pointed out that the degree of tumor regression might be a very important clinical tool, as it could be used as a prognostic marker; however, the clinical significance of tumor pathological response is still under investigation [7,27]. In our cohort, pathological response is not associated with overall survival (OS) and disease-free survival (DFS). However, we showed that miR-31 deregulation is able to predict pathological response, DFS, and OS. In this way, 78% of the patients with high miR-31 expression were alive at 6 years follow up compared with 96% of those with low miR-31. In addition, 65% of the patients with high miR-31 had a recurrence within the first three years of follow up, compared with 86% of the patients with low miR-31. Those results suggest the strength of miR-31 as a predictive biomarker to guide multidisciplinary treatment in patients with LARC, since cases with high miR-31 had no appreciable clinical benefit from neoadjuvant CRT. Considering these results, high miR-31 patients could benefit from an alternative therapeutic approach, different than 5-FU-based neoadjuvant CRT.

Measuring miR-31 expression levels as a predictive biomarker for neoadjuvant CRT in LARC has the advantage that is easily detected from small amounts of routinely-prepared formalin-fixed paraffin-embedded (FFPE) endoscopic samples by using a RT-PCR. Furthermore, microRNAs have the ability to continue stable, even when subjected to extreme conditions such as very low or high pH levels, boiling, longer storage time, and multiple freeze-thaw cycles [28]. Limitations of our study include the low number of cases included, the lack of validation in an independent larger cohort, and that it was a retrospective study instead of a prospective one. Therefore, the conclusions provided by this study have to be interpreted with caution and the potential clinical usefulness of miR-31 has to be further confirmed in forthcoming studies and controlled randomized clinical trials before a potential inclusion in clinical protocols. However, it is also true that this study is larger than other studies evaluating neoadjuvant CRT treatments, and the treatment regimen was very homogeneous.

Finally, prior works have described the interaction of miR-31 with important tumor suppressor genes, such as the enhancer of zeste homolog 2 (EZH2) [29] and the hypoxia-inducible transcription factor 1α (HIF-1α) [30]. In the same way, in other tumor models, miR-31 represses the regulatory subunit B alpha isoform of the tumor suppressor PP2A (PPP2R2A) [31]. We previously reported that this subunit downregulation is a common event in colorectal cancer patients and its relation with the resistance to 5-FU [32]. According to this and despite there are likely to be multiple explanations for primary or acquired resistance to neoadjuvant CRT—exploring the role and interaction of miR-31 and PPP2R2A to predict neoadjuvant CRT response in LARC patients could be of great interest for future research.

## 4. Materials and Methods

### 4.1. Patient Selection

Eighty-one patients diagnosed with LARC and treated with neoadjuvant CRT between the beginning of 2007 and the end of 2012 (6 years) at the University Hospital Fundación Jiménez Díaz, (Madrid, Spain) were retrospectively selected and included in this study. They were followed-up until February 2016.

All patients had a preoperative staging based on transrectal ultrasound (TRUS) and/or magnetic resonance image (MRI) of the pelvis. In addition, a full body computed tomography scan (FBCT) was performed to exclude stage IV disease. The patients were treated with rule-based chemoradiotherapy regimens based on 5-FU and underwent surgery after one and a half to two months after neoadjuvant CRT completion. Every patient gave written informed consent for tissue storage and analysis at Fundación Jiménez Díaz biobank, Madrid (Spain). Fundación Jiménez Díaz University Hospital, Madrid (Spain) institutional review board approved the study.

### 4.2. Pathologic Response and Tumor Samples

The Fundación Jimenez Diaz biobank provided the tumor samples. All tissue derived from the surgical resection were classified in concordance with the College of American Pathologist guidelines for invasive carcinomas (TNM, 7th ed.). Two independent pathologists blinded to all patients’ clinical data evaluated tumor regression grade according to the modified Ryan classification that categorizes tumors in four levels of response: complete, moderate, minimal, and poor response. Complete response score 0 indicates no viable cancer cells; moderate score 1 indicates single cells or little groups of cancer cells; minimal score 2 indicates residual cancer outgrown by fibrosis; and poor response scores 3 indicates minimal or no tumor kill with extensive residual cancer. Every regression grade was compared with the primary tumor, in concordance with clinical guidelines [33].

### 4.3. RNA Isolation

We isolated total RNA from formalin-fixed paraffin-embedded (FFPE) tumor biopsies, applying RecoverAll Total Nucleic Acid Isolation kit Ambion (Thermo Fisher Scientific, USA in accordance with manufacturer’s instructions. We eluted total RNA and quantified it with a NanoDrop Spectrophotometer (Thermo scientific, USA)

### 4.4. Quantification of MicroRNA Expression Levels

Samples were reverse transcribed using the TaqManHMicroRNA Reverse Transcription Kit (Applied Biosystems Waltham, MA, USA). Working with TaqMan MicroRNA Assays (Applied Biosystems, USA) specific for miR-31 (reference number: 002279) and U6B as internal control, mature miRNAs were quantified by quantitative real-time reverse transcription polymerase chain reaction (RT-PCR). TaqMan MicroRNA assays have an ultra-high sensitivity and can detect as few as ten copies of the target transcript in a sample and also have a dynamic range greater than seven logs. Reactions were carried out using an Applied Biosystems 7500 Sequence Detection System. Conditions: 95 °C for 10 min, followed by 45 cycles of 95 °C for 15 s, and 60 °C for 1 min. The Δ*C*_T_ method was performed to analyze relative gene expression data.

### 4.5. Statistical Analysis

We used SPSS v14.0 (SPSS Inc, Chicago, IL, USA) for statistical analyses. We applied the χ2 test (Fisher exact test) based on bimodal distribution of data to evaluate the correlation between miR-31 overexpression and the clinical and pathological variables. All reported *p* values are two-sided. We made a receiver operating characteristic (ROC) curve to establish a cutoff for miR-31 expression, calculated its 95% confidence interval, and selected the cutoff point that provides us with the best specificity and sensitivity to differentiate rectal cancer pathological response, in order to assess the potential usefulness of miR-31 as a predictive biomarker. According to this criteria, we considered up-regulation when miR-31 expression levels (Δ*C*_T_) were lower than 0.34. We defined DFS as the time from surgery until recurrence, appearance of a secondary tumor, or death, and OS as the time from the date of diagnosis to the date of last follow-up or death, and applied the Kaplan-Meier method and survival comparisons with the log-rank test and Breslow. Then, we adjusted the Cox proportional hazards model by taking into consideration significant parameters in univariate analysis and considered a *p* value <0.05 statistically significant. We followed the Reporting Recommendations for Tumor Marker Prognostic Studies (REMARK) guideline [34].

## 5. Conclusions

In conclusion, our work shows evidence for the potential utility of miR-31 as a predictive biomarker for pathological response and outcome in patients with LARC treated with neoadjuvant CRT—a concept that can be very interesting to better select treatment options in patients with LARC and that can be incorporated into daily clinical practice. However, more studies are needed to better define the biological and clinical significance of miR-31 in rectal cancer. Furthermore, a validation of these results in a larger independent LARC patient cohort is necessary to validate our results.

## Figures and Tables

**Table 1:** Baseline clinical and pathological characteristics of our locally advanced rectal cancer (LARC) cohort and its relation with miR-31 expression.

Clinical and pathological characteristics		nº Cases (%)	nº miR-31 High ^1^ (%)	nº miR-31 Low ^2^ (%)	*p*
miR-31 ^3 pre^ CRT		78 (100%)	27 (34.2)	51 (65.8)	0.267
Age	<60	24 (30.7)	10 (41.6)	14 (58,4)	
	>60	54 (69.3)	17 (31.4)	37 (68.6)	
Sex	Male	47 (60)	15 (31.9)	32 (68.1)	0.454
	Female	31 (40)	8 (68.1)	19 (31.9)	
ECOG ^4^	0	49 (62.8)	14 (28.5)	35 (71.5)	0.113
	I	29 (37.2)	13 (71.5)	16 (28.5)	
Clinical stage pre CRT ^5^	II	4 (5.2)	3 (75)	1 (25)	0.121
	III	73 (94.8)	24 (30.7)	49 (69.3)	
Neoadjuvant CRT	RT + 5-FU ^6^ based	78 (100%)			
Adjuvant therapy	5-FU	55 (70.5)	20 (36.4)	35 (63.6)	0.407
	FOLFOX ^7^	6 (7.7)	3 (50)	3 (50)	
	Other	17 (21.8)	4 (23.5)	13 (76.5)	
Grade pre CRT	Low	20 (25.8)	5 (25)	15 (75)	0.410
	High	50 (64)	20 (40)	30 (60)	
	ND ^8^	8 (10.2)	2 (25)	6 (75)	
ypT ^9^	ypT0	10 (12.8)	1 (10)	9 (90)	0.372
	ypT1-2	33 (42.4)	13 (39.4)	20 (60.6)	
	ypT3-4	32 (41)	12 (37.5)	20 (62.5)	
	ypTx	3 (3.8)	1 (33.3)	2 (66.7)	
ypN ^10^	pN0	61 (78.2)	18 (29.5)	43 (70.5)	0.086
	pN+	17 (21.8)	9 (52.9)	8 (47.1)	
Pathological stage	ypT0N0	10 (12.8)	1 (10)	9 (90)	0.133
	ypI	30 (38.5)	11 (36.6)	19 (63.4)	
	ypII	21 (27)	6 (28.6)	15 (71.4)	
	ypIII	17 (21.7)	9 (52.9)	8 (47.1)	
Downstaging	No	19 (24.3)	9 (47.4)	10 (52.6)	0.143
	Yes	59 (75.7)	18 (52.6)	41 (47.4)	
Pathology response	Complete response	10 (12.8)	1 (10)	9 (90)	0.018
	Moderate response	30 (38.5)	7 (23.3)	23 (76.7)	
	Minimal response	15 (19.2)	5 (33.3)	10 (66.7)	
	Poor response	23 (29.5)	14 (60.8)	9 (39.2)	

^1^ miR-31 high = Δ*C*_T_ miR-31 < 0.34 (cut-off established by a receiver operating characteristic (ROC) curve); ^2^ miR-31 low = Δ*C*_T_ miR-31 > 0.34 (cut-off established using a ROC curve); ^3^ miR-31 = microRNA 31; ^4^ ECOG = Eastern Cooperative Oncology Group; ^5^ CRT = Chemoradiotherapy; ^6^ 5-FU = 5-fluorouracil; ^7^ FOLFOX = Fluorouracil, Oxaliplatin, Leucovorin; ^8^ ND = No data; ^9^ ypT = tumor size after CRT; ^10^ ypN = pathological lymph node after CRT.

**Table 2:** MiR-31 predicts pathological response to neoadjuvant CRT.

Responders *vs.* Non-Responders			
Response	Response ^1^	Non-Response ^2^	Total
miR-31 high ^3^	13	14	27
miR-31 low ^4^	42	9	51
Total	55	23	78
NPV ^5^ (%) 82.3		Specificity (%) 76.3	
PPV ^6^ (%) 51.8		Sensitivity (%) 60.8	

^1^ “Response” = moderate, minimal, or complete response; ^2^ “Non-Response” = poor pathological response; ^3^ miR-31 high = Δ*C*_T_ miR-31 < 0.34 (cutoff derived by receive operating characteristic curve); ^4^ miR-31 low= Δ*C*_T_ miR-31 > 0.34 (cutoff derived by receive operating characteristic curve); ^5^ NPV = negative predictive value; ^6^ PPV = positive predictive value.

**Table 3:** Multivariable logistic analysis for miR-31 expression measured pre neoadjuvant CRT and the rest of clinic-pathological factors measured before neoadjuvant CRT in responder and non-responder patients.

Responders ^1^ *vs.* Non-Responders ^2^	^3^ OR (95% CI ^4^)	*p*
Age, <60 *vs.* >60	0.97 (0.30 to 3.06)	0.962
Gender, Female *vs.* Male	1.25 (0.41 to 3.86)	0.452
ECOG ^5^, O *vs.* I	0.79 (0.25 to 2.51)	0.799
Grade pre CRT ^6^, high-moderate *vs.* low	1.25 (0.53 to 2.96)	0.600
Clinical stage, II *vs.* III	2.51 (0.20 to 30.37)	0.468
miR-31 high ^7^ *vs.* low ^8^	0.18 (0.06 to 0.57)	0.003

^1^ “*Response*” = moderate, minimal or complete response; ^2^ “*Non-Response*” = poor pathological response; ^3^ OR = Odds ratio; ^4^ CI = Confidence interval; ^5^ ECOG = Eastern Cooperative Oncology Group; ^6^ CRT= chemoradiotherapy; ^7^ miR-31 high = Δ*C*_T_ miR-31 < 0.34 (cutoff derived by receive operating characteristic curve); ^8^ miR-31 low= Δ*C*_T_ miR-31 > 0.34 (cutoff obtained by a receive operating characteristic curve).

**Table 4:** LARC patients factors predictive of poor overall survival: Univariate and multivariable analyses.

Variables	Univariate		Multivariate	
	HR ^1^ (95% CI ^2^)	*p*	HR (95% CI)	*p*
Age, >60 *vs.* <60	0.915 (0.246 to 3.407)	0.895		
Sex, female *vs.* male	1.024 (0.56 to 1.86)	0.928		
Clinical stage, II *vs.* III	2.229 (0.274 to 18.132)	0.453		
Pathological ypT ^3^, ypT0 *vs.* ypT1/T2 *vs.* ypT3/T4	2.064 (0.214 to 19.902)	0.531		
Pathological ypN ^4^, N+ *vs.* N−	2.907 (0.918 to 9.211)	0.070		
Pathological stage	1.890 (1.027 to 3.478)	0.043	2.411 (1.136 to 5.114)	0.022
Pathological response, poor *vs.* complete *vs.* moderate *vs.* minimal	1.346 (0.70 to 2.56)	0.366		
MiR-31 expression, high ^5^ (Δ*C*_T_ < 0.34) *vs.* low ^6^ (Δ*C*_T_ > 0.34)	5.077 (1.366 to 18.863)	0.015	0.206 (0.051 to 0.840)	0.028

^1^ HR = Hazard ratio; ^2^ CI = Confidence Interval; ^3^ ypT = tumor size after CRT; ^4^ ypN = pathological lymph node after CRT; ^5^ miR-31 high = Δ*C*_T_ miR-31 < 0.34 (cutoff derived by receive operating characteristic curve); ^6^ miR-31 low = Δ*C*_T_ miR-31 > 0.34 (cutoff derived by receive operating characteristic curve).

## Abbreviations

LARC	Locally advanced rectal cancer
CRT	Chemoradiotherapy
MiR	MicroRNA
MiR-31	MicroRNA-31
FFPE	formalin-fixed paraffin-embedded
5-FU	5-fluorouracil
pCR	pathological complete response; ypT0N0
CRC	colorectal cancer
anti-EGFR	anti-epithelial growth factor receptor
RT-PCR	quantitative real-time polymerase chain reaction
PPP2R2A	regulatory subunit B alpha isoform of the tumor suppressor PP2A
MRI	Magnetic resonance image
TRUS	Transrectal ultrasound
FBCT	full body computed tomography scan
REMARK	Reporting Recommendations for Tumor Marker Prognostic Studies
